# A prospective cohort study of the risk factors for new falls and fragility fractures in self-caring elderly patients aged 80 years and over

**DOI:** 10.1186/s12877-021-02043-x

**Published:** 2021-02-10

**Authors:** Jian Zhou, Bo Liu, Ming-Zhao Qin, Jin-Ping Liu

**Affiliations:** 1 Department of Geriatrics, Beijing Tongren Hospital, Capital Medical University, Beijing100730, China. No.1 of Dong Jiao Min Xiang, Dongcheng District, Beijing, 100730 China; 2 Department of Otolaryngology Head and Neck Surgery, Beijing Tongren Hospital, Capital Medical University, Beijing Institute of Otolaryngology, Key Laboratory of Otolaryngology Head and Neck Surgery(Capital Medical University) , Ministry of Education, Beijing100730, China. No.1 of Dong Jiao Min Xiang, Dongcheng District, Beijing, 100730 China

**Keywords:** Falls, Fragility fractures, Aged 80 years and over, Timed up-and-go (TUG) test, Walking speed

## Abstract

**Background:**

This study aimed to prospectively analyze the risk factors for new falls and fragility fractures in self-caring elderly patients and to find suitable evaluation tools for community screening and follow-up interventions. **Methods:** A total of 300 participants (187 male and 113 female), aged 80 or above and capable of caring for themselves, were enrolled in this study and observed for a period of 12 months. Their medical histories were collected, various indicators were measured, and the risk factors for new falls and fragility fractures were analyzed.

**Results:**

A total of 290 participants were included in the statistical analysis. Eighty-seven participants (30%) had new falls. The incidence was negatively correlated with the activities of daily living (ADL, represented by the Barthel Index) score (*P*=0.008) but was positively correlated with the timed up-and-go (TUG) test score> 12 s (*P*=0.021). The results also revealed that 33 fragility fractures occurred in 29 patients (10.0%), which was positively correlated with new falls (*P*=0.000). New fragility fractures were negatively correlated with the bone mineral density (BMD) of the lumbar vertebrae (*P*=0.012) and walking speed (*P*=0.000).

**Conclusion:**

TUG, walking speed, the ADL score, and the fall risk assessment scale can simply and effectively assess the risk of new falls and fragility fractures in the elderly population, and their use should be widely implemented in the community.

## Background

The type of fall this study is looking at is one in which a person loses their balance when they are standing or upright and, as a result, they end up on the ground or at a lower level. Other types of fall such as those caused by an act of violence, loss of consciousness, hemiplegia or epileptic seizure are not included in this study [1]. For the elderly population, a fall is an independent risk factor of fragility fracture. In other words, its consequences can be serious [1]. Falls in the elderly population are the result of the combined action of internal factors, such as the state of their health, and external factors, such as their environment [2]. The elderly are subject to the natural deterioration of tissue structure and the physiological function of various systems, which can result, in particular, in lower limb weakness. This is an important risk factor for the occurrence of falls in the elderly. Gait disorder, walking instability, and balance impairment are caused by a decline in the structure and functioning of the central nervous system and peripheral nerves, which is also closely related to diseases such as spinal stenosis and osteoarthritis [2]. The impairment of vision, hearing, muscle strength, and response speed will also reduce the ability of elderly patients to respond to stimuli, and they will also experience the loss of their self-protection ability, further increasing the risk of falls and injury [3]. Multi-system complications of the elderly, such as cardiovascular and cerebrovascular diseases, diabetes, mental disorders, cataracts, hearing impairment, and the long-term use of a variety of drugs are also risk factors for falls in this population [3]. In turn, fall-induced injuries increase the risk of delirium, cognitive decline and disability in the elderly.

Falling has a high incidence rate in the elderly population nowadays, and it has become a public health problem attracting great concern. Studies have revealed that 40–50% of the elderly aged ≥80, living in the community, fall at least once a year [2, 3] and that 90% of fragility fractures in patients with osteoporosis are caused by falls [4]. An osteoporotic fracture, which is regarded as a low-energy fracture [5], is seen as a non-traumatic or slight traumatic fracture and is a clear sign of the reduction in bone strength, and, thus, an eventual consequence of osteoporosis. The common sites of such fractures are the spine, hip, and distal forearm. Fragility fractures result in a heavy economic burden on patients and their families, as well as on society as a whole. As the population structure changes, and life expectancy increases, this burden is expected to increase in the future [6]. China has officially been an aging society since 2000, and it is estimated that the number of osteoporosis patients in China will have increased from 83.9 million in 1997 to 212 million by 2050 [7, 8]. Approximately 2.33 million osteoporotic fractures were estimated to have occurred in 2010, costing $9.45 billion. However, it has been estimated that the annual number and costs of osteoporosis-related fractures will increase to 5.99 million fractures, costing $25.43 billion, by 2050 [9]. The researchers in a previous cross-section survey found that 47.7% of fragility fracture patients aged 80 years and over had osteoporosis, 40.9% of them had osteopenia, while only 11.4% of them had normal bone mass. The risk factors of fragility fracture were found to be falls and lumbar bone mineral density (BMD) reduction [10].

The prevention of fragility fractures has shifted from simple anti-osteoporosis treatment to a combination of treatment and fall prevention action, and the importance of the latter is gaining more attention [4]. However, it is very difficult for clinicians to screen and follow-up with the elderly in the community. People’s awareness of osteoporosis is also poor, and its harm to the human body is not understood, so the treatment rate is low, and many patients are not diagnosed until they have a fracture. This leads to an increase in the incidence of fragility fractures. Therefore, in the present study, the risk factors of new falls and fragility fractures in self-caring elderly patients aged 80 years and over were prospectively analyzed. Initial screening evaluation tools suitable for use in the community were recommended, and high-risk persons were screened ahead of time. The researchers cooperated with hospitals to further analyze the underlying causes and carry out early intervention and follow-up, so as to effectively extend the life and self-care time of the elderly while at the same time reducing the social and economic costs involved [6, 11].

## Methods

### Subjects

The inclusion criteria were as follows: patients aged ≥80 years and above who were able to take care of themselves. The exclusion criteria were as follows: patients with hyperthyroidism or hypothyroidism, primary hyperparathyroidism or hypothyroidism, Cushing’s syndrome, osteomalacia, malignant tumor, chronic kidney disease (stage 5), cirrhosis, or chronic obstructive pulmonary disease (COPD) (grade 4).

Between January and June 2018, 300 eligible patients from the Outpatients Department of Geriatrics of Beijing Tongren Hospital were enrolled in the study, having given their consent, and each person was monitored for 12 months.

### Medical history collection and risk evaluation

The present study adopted a prospective cohort study design. Any new falls and new fragility fractures in these patients during the 12-month follow-up period were recorded. Baseline data were collected either by means of self-reporting or carer-reporting, and through a review of the patient’s medical history and imaging results. During the study, data were collected by the researcher via the outpatient visits of the participants, or via telephone contact. When there was a new fall, and the X-ray showed compressive changes in the thoracolumbar spine, the participants would go to the orthopedics department to obtain confirmation of a fragility fracture.

The patient’s medical history (consultation and review of previous medical records) and physical examination results were collected by specially trained doctors at an osteoporosis and falls clinic. The ability to cope with daily living activities was assessed by the Activity of Daily Living (ADL) scale (Barthel Index), which is scored as follows: 100 points =ADL complete self-care; 75–95 points = mild functional impairment; 50–70 points = moderate functional impairment; 25–45 points = severe functional impairment; 0–20 points = very serious functional impairment. A score of ≥75 was defined as being capable of basic self-care [12]. The fall risk assessment (FRA) scale was used for rating fall risk: low risk 1–2 points, medium risk 3–9 points, and high risk ≥10 points [13]. The height and weight of patients were measured by a specially trained nurse. Muscle strength was measured using the grip strength method, whereby the grip strength of the subjects was measured with a Jamar hand grip (Sammons Preston, USA), and the patients’ hands were tested twice each, the higher value being recorded. A male grip strength of < 30 kg and a female one of < 20 kg was regarded as showing decreased muscle strength [14]. The muscle function was evaluated using the usual gait speed (UGS). Patients were tested by a step speed test over a distance of 6 m, and a walking speed of < 0.8 m/s was defined as showing decreased walking ability. For the timed up & go (TUG) test, the subjects stood up from a seat with an armrest at a normal height (the seat height was approximately 48 cm and the armrest height was approximately 68 cm), walked at their normal walking speed for 3 m, then turned, returned and sat down again, and the time from standing up to sitting down again was recorded [15]. A TUG result of > 12 s was defined as having a high risk of falls [16]. Finally, the chair rising test (CRT) consisted of the subjects standing up and sitting down again five times from a chair of normal height (the height of the seat was approximately 48 cm). The time from first standing up to the 5th time they sat down and touched the chair was recorded. A CRT result of > 10 s or < 5 times was defined as representing a high fall risk [17].

### Determination of the biochemical indexes

Between 8 and 10 am, 10 ml of elbow vein fasting blood was collected and immediately sent to the laboratory to test the patient’s renal function [blood urea nitrogen (BUN), creatinine (Cr), albumin (ALB)], electrolytes (Ca^2+^ and inorganic phosphorus), intact parathyroid hormone (PTH), total testosterone (T), and estradiol (E2), using a Beckman CX4CE automatic biochemical analyzer. The type I amino-terminal propeptide (P1NP), β-C-terminal telopeptide of type I collagen (β-CTx), osteocalcin (OC), and 25-hydroxyvitamin (25OHD) were detected with a Japanese Rochee 60 L immunoluminescent analyzer, using electrochemical luminescence, the kit being produced by Roche Diagnostics (Germany). The serum calcium level was the serum calcium (mmol/L) corrected by albumin = serum calcium (mmol/L) + (0.8–0.02 × albumin [g/L]). The eGFR level was calculated according to the MDRD formula.

### BMD and X-ray scans

The BMD and T-score of lumbar 1–4, bilateral femoral neck and total hip were measured with a dual-energy X-ray absorptiometry (DXA) produced by GE (USA). The diagnosis was made based on the lowest T-value, according to the World Health Organization (WHO) diagnostic criteria, which are as follows: Normal bone mass T-value≥− 1; osteopenia − 2.5<T-value<− 1; and, osteoporosis T-value ≤− 2.5 [5]. Patients with obvious lower back pain or a decrease in height of ≥4 cm had to undergo a non-enhanced lateral X-ray scan of the thoracolumbar spine. During the observation period, if there was a new fall or severe back pain, the thoracolumbar XR was examined, and if there was compression of the vertebral body, the patient was referred to the orthopedic department for further diagnosis of a new or old fracture.

### Statistical analysis

Data were statistically analyzed using SPSS18.0 software. The test of normality of measurement data was carried out first. Normally distributed measurement data were expressed as mean ± standard deviation, and the measurement data that were normally distributed after the log conversion were expressed as mean (95% confidence interval). The means were compared using two independent sample t-tests. Non-normally distributed data were expressed as the median (interquartile range) and compared using a nonparametric test. Count data were expressed as a percentage and compared using a Chi-square test. The analysis of related factors was carried out using binary logistic regression. *P*< 0.05 was considered statistically significant.

## Results

### Basic information

The average age of these patients was 83.92 ± 3.28 years, and 187 patients (62.3%) were male and 113 patients (37.7%) were female. The ratio of M:F=1.65:1 as women were less willing than men to participate in the study. The patients were all residents of Beijing.

During the 12-month observational period, ten of the 300 patients initially selected stopped participating in the study; four patients broke off contact with the investigators and six of them died, the cause of death of three of them being severe pneumonia and that of the remaining patients being lung cancer, sudden cardiac death, and cerebral hemorrhage. In the end, a total of 290 persons were included in the statistical analysis.

### A comparison of new falls among populations with different characteristics and the related factors of new falls

In the present study, 87 persons had new falls, the incidence being 30% (87/290). It was found that people that took part in outdoor activities of < 30 min/day, people who were older than 80 years of age, people with diabetes mellitus, people with a walking speed of < 0.8 m/s, and people with a TUG result of > 12 s were more likely to have new falls, and the difference was statistically significant (*P*< 0.05, *P*< 0.01, Table 1). Compared with patients who did not have any new falls, these patients had a lower ADL (Barthel Index) score, higher FRA score, lower testosterone level, lower grip strength, lower walking speed, lower right femoral neck and total hip BMD, and longer TUG and CRT, the differences being statistically significant (*P*< 0.05, *P*< 0.01). See Table 2.

**Table 1:** The comparison of new falls and fractures in populations with different characteristics

Item		Number of cases n(%)	Number of new falls n(%)	*p*	New brittle fractures n(%)	*p*
Gender	Male	180 (62.1%)	48 (26.7%)	0.116	12 (6.7%)	0.016
	Female	110 (37.9%)	39 (35.5%)		17 (15.5%)	
Outdoor activities	< 30 min/d	80 (27.6%)	32 (40.0%)	0.048	14 (17.5%)	0.031
	30 min-1 h/d	64 (22.1%)	14 (21.9%)		5 (7.8%)	
	> 1 h/d	146 (50.3%)	41 (28.1%)		10 (6.8%)	
Falls occurring after 80	Yes	155 (53.4%)	56 (36.1%)	0.015	17 (11.0%)	0.556
	No	135 (46.6%)	31 (23.0%)		12 (8.9%)	
The history of fracture	Yes	72 (24.8%)	27 (37.5%)	0.109	13 (18.1%)	0.009
	No	218 (75.2%)	60 (27.5%)		16 (7.3%)	
Coronary heart disease	Yes	133 (45.9%)	45 (33.8%)	0.190	11 (8.3%)	0.366
	No	157 (54.1%)	42 (26.8%)		18 (11.5%)	
Cerebral blood Tube disease	Yes	109 (37.6%)	36 (33.0%)	0.383	12 (11.0%)	0.657
	No	181 (62.4%)	51 (28.2%)		17 (9.4%)	
Diabetes	Yes	89 (30.7%)	34 (38.2%)	0.043	10 (11.2%)	0.641
	No	201 (69.3%)	53 (26.4%)		19 (9.5%)	
Chronic kidney disease	Stage 1	57 (19.7%)	21 (36.8%)	0.444	8 (14.0%)	0.252
	Stage 2	160 (55.2%)	46 (28.8%)		17 (10.6%)	
	Stage 3–4	73 (25.2%)	20 (27.4%)		4 (5.5%)	
Osteoporosis	Normal bone mass	78 (26.9%)	18 (23.1%)	0.229	4 (5.1%)	0.221
	Low bone mass	127 (43.8%)	39 (30.7%)		14 (11.0%)	
	Osteoporosis	85 (29.3%)	30 (35.3%)		11 (12.9%)	
Walking speed_2_	>=0.8 m/s	170 (66.4%)	42 (24.7%)	0.014	9 (5.3%)	0.002
	< 0.8 m/s	86 (33.6%)	34 (39.5%)		15 (17.4%)	
TUG	<=12 s	121 (47.3%)	25 (20.7%)	0.003	3 (2.5%)	0.000
	> 12 s	135 (52.7%)	51 (37.8%)		21 (15.6%)	
CRT	<=10 s	35 (13.7%)	9 (25.7%)	0.580	2 (5.7%)	0.424
	> 10 s	221 (86.3%)	67 (30.3%)		22 (10.0%)	
Treatment plan	Ca^2+^/ VitD	46 (15.9%)	12 (26.1%)	0.220	2 (4.3%)	0.318
	Ca^2+^+ Active VitD	123 (42.4%)	32 (26.0%)		15 (12.2%)	
	Ca^2+^+ Active VitD+anti-osteoporosis drugs	121 (41.7%)	43 (35.5%)		12 (9.9%)	
New falls	Yes	87 (30.0%)			24 (27.6%)	0.000
	No	203 (70.0%)			5 (2.5%)	
New brittle fractures	Yes	29 (10.0%)	24 (82.8%)	0.000		
	No	261 (90.0%)	63 (24.1%)			
Total		290	87		29	

*TUG* timed up & go test, *CRT* chair rising test, History of falls:falls occurring after 80 years old

**Table 2:** The comparison of the data in patients with new falls and without falls

Items	With new falls(*n*=87)	Without new fall experiences(*n*=203)	*P*
Age (years)	84.4±3.5	83.6±3.1	0.058
BMI (kg/m^2^)	23.2±3.2	23.7±3.0	0.247
ADL (score)	95 (95, 100)	100 (95, 100)	0.001
FRA (score)	10 (10, 12)	10 (8, 11)	0.000
Ca^2+^ (mmol/L)	2.31±0.10	2.33±0.09	0.122
Inorganic phosphorus (mmol/L)	1.11±0.16	1.11±0.16	0.773
PTH (pg/mL)	44.21 (39.79~48.85)	44.31 (40.94~47.65)	0.411
E_2_ (pg/mL)	30.36±19.19	31.86±19.08	0.547
T (ng/mL)	1.93 (0.29, 3.01)	2.57 (0.33, 3.77)	0.022
25OHD (ng/mL)	20.32±9.26	21.76±11.85	0.313
eGFR (mL/min)	75.98±22.32	72.70±19.36	0.209
OC (ng/mL)	12.47 (11.36~13.90)	13.14 (12.35~13.90)	0.251
P1NP (ng/mL)	31.99 (29.54~34.78)	31.48 (29.29~33.87)	0.410
β-CTx (ng/mL)	0.18 (0.13, 0.29)	0.21 (0.14,0.30)	0.216
L_1-4_BMD (g/cm^2^)	1.18±0.31	1.16±0.25	0.611
LnBMD (g/cm^2^)	0.76±0.15	0.79±0.16	0.167
RnBMD (g/cm^2^)	0.75±0.14	0.80±0.16	0.015
LtBMD (g/cm^2^)	0.84±0.16	0.86±0.17	0.308
RtBMD (g/cm^2^)	0.82±0.16	0.86±0.17	0.032
Grip (kg)	24.51±6.93	27.18±8.95	0.010
Walking speed (m/s)	0.84±0.24	0.96±0.25	0.001
TUG (s)	15.43 (13.78~17.48)	13.16 (12.48~13.94)	0.009
CRT (s)	16.32 (14.90~17.88)	14.65 (13.88~15.46)	0.027

*BMI* Body Mass Index, *ADL* Activity of Daily Living scale, *FRA* The fall risk assessment, *PTH* parathyroid hormone, *E*_*2*_ estradiol, *T* Total testosterone, *25(OH)D* 25-hydroxyvitamin D, *eGFR* glomerular filtration rate, *OC* osteocalcin, *P1NP* type I amino-terminal propeptide, *β-CTx* β-C-terminal telopeptide of type I collagen, *L*_*1-4*_*BMD* bone mineral density of lumbar, *LnBMD* bone mineral density of left femoral neck, *RnBMD* bone mineral density of right femoral neck, *LtBMD* bone mineral density of left total hip, *RtBMD* bone mineral density of right total hip, *TUG* timed up & go, *CRT* chair rising test

An analysis of the related factors revealed that the ADL (Barthel Index) score was negatively correlated with new falls (OR=0.911 [0.850–0.976], *P*=0.008), while the TUG results of > 12 s were positively correlated with new falls (OR=1.980 [1.109–3.532], *P*=0.021). See Table 3.

**Table 3:** The related factors of new falls

Factor	*B*	*S.E.*	*Wald*	*P*	*OR (95%CI)*
ADL	−0.093	0.035	6.933	0.008	0.911 (0.850–0.976)
TUG_2_	0.683	0.295	5.342	0.021	1.980 (1.109–3.532)
Constant	7.143	3.567	4.010	0.045	1264.667

*ADL* Activity of Daily Living, *TUG*_*2*_ TUG>12 s

### A comparison of new fragility fractures among populations with different characteristics and related factors of new fragility fractures

In the present study, 33 fragility fractures occurred in 29 patients (10.0%), the incidence being 10.0% (29/290). The fracture sites were the thoracolumbar vertebrae (42.4%, 14/33), hip (30.3%, 10/33), distal forearm (12.1%, 4/33), and other body parts (ribs, clavicles, elbows, and skull) (15.2%, 5/33).

Among these patients, the female patients with outdoor activities of < 30 min/day, a history of fragility fractures, a walking speed of < 0.8 m/s, a TUG result of > 12 s, and new falls were more likely to have new fragility fractures, the difference being statistically significant (*P*< 0.05, *P*< 0.01). See Table 1. Compared with patients without new fragility fractures, these patients had a higher FRA score, lower grip strength, lower walking speed, lower right femoral neck and total hip BMD, longer TUG and CRT, and the differences were statistically significant (P< 0.05, P< 0.01). See Table 4.

**Table 4:** The comparison of the data in patients with new fractures and without fractures

Items	With new brittle fractures(*n*=29)	Without new brittle fractures(*n*=261)	*P*
Age (years)	84.9±2.9	83.8±3.3	0.078
BMI (kg/m^2^)	22.9±3.5	23.6±3.0	0.242
ADL (score)	100 (90, 100)	100 (95, 100)	0.288
FRA (score)	11 (10, 14)	10 (8, 11)	0.003
Ca^2+^	2.30±0.10	2.33±0.09	0.157
Inorganic phosphorus (mmol/L)	1.11±0.15	1.11±0.16	0.916
PTH (pg/mL)	42.35 (36.40~48.77)	44.48 (41.46~47.34)	0.721
E_2_ (pg/mL)	30.85±20.53	31.47±18.98	0.874
T (ng/mL)	0.59 (0.23, 3.13)	2.33 (0.33, 3.58)	0.183
25OHD (ng/mL)	22.70±7.57	21.18±11.47	0.485
eGFR (mL/min)	79.24±27.03	73.07±19.39	0.121
OC (ng/mL)	11.71 (9.96~13.58)	13.07 (12.40~13.81)	0.229
P1NP (ng/mL)	30.64 (26.35~34.90)	31.73 (29.84~33.70)	0.905
β-CTx (ng/mL)	0.19 (0.13, 0.25)	0.20 (0.14, 0.29)	0.542
L_1-4_BMD (g/cm^2^)	1.04±0.23	1.18±0.27	0.009
LnBMD (g/cm^2^)	0.72±0.16	0.79±0.16	0.030
RnBMD (g/cm^2^)	0.72±0.12	0.79±0.16	0.015
LtBMD (g/cm^2^)	0.80±0.14	0.86±0.17	0.089
RtBMD (g/cm^2^)	0.78±0.14	0.86±0.17	0.013
Grip (kg)	21.14±6.78	26.95±8.45	0.001
Walking speed (m/s)	0.70±0.19	0.95±0.25	0.000
TUG (s)	17.61 (14.96~20.80)	13.45 (12.74~14.28)	0.000
CRT (s)	18.25 (15.45~21.31)	14.83 (14.12~15.59)	0.005

An analysis of the related factors revealed that new falls were significantly positively correlated with new fragility fractures (OR=11.885 [3.914–36.087], *P*=0.000). The BMD of lumbar vertebrae and walking speed were significantly negatively correlated with new fragility fractures, (OR=0.092 [0.014–0.596], *P*=0.012, OR=0.011 [0.001–0.124], P=0.000, respectively). See Table 5.

**Table 5:** The related factors of new fractures

Factor	*B*	*S.E.*	*Wald*	*P*	*OR (95%CI)*
New falls	2.475	0.567	19.080	0.000	11.885 (3.914–36.087)
L_1-4_BMD	−2.385	0.953	6.261	0.012	0.092 (0.014–0.596)
Walking speed	−4.521	1.241	13.264	0.000	0.011 (0.001–0.124)
Constant	2.731	1.453	3.535	0.060	15.345

## Discussion

The elderly population has a high risk of falls and needs to receive assessment of fall-related risk factors and intervention when required [18]. The present study involved people aged 80 years and over who could care for themselves in the community. Their daily activities, underlying diseases, biochemical indexes, 25(OH)D, sex hormones, parathyroid hormone levels (PTH), bone turnover markers, BMD, muscle strength, muscle function, balance function, and use of anti-osteoporosis drugs were analyzed and prospectively observed for 12 months.

This study revealed that compared with patients who did not have falls, patients with new falls had a higher FRA score, lower ADL (Barthel Index) score, lower testosterone, lower BMD of the femoral neck and total hip, lower grip strength, lower walking speed, and longer TUG and CRT. The logistic regression analysis further revealed that the low ADL (Barthel index) score and TUG of > 12 s were the risk factors of new falls in the elderly with a basic self-care ability. The TUG test is a method developed by the McGill University of Canada to test functional mobility [19]. In clinical practice, it is believed that this method can be used to simply and reliably evaluate patients’ ability to balance, their coordination function, joint range of motion, and reflex control, and, thus, it is used to predict fall risk [20, 21]. It is also an independent predictor of non-vertebral and hip fractures [20]. The American and British Geriatric Societies [22] and the National Institute of Clinical Evidence (NICE) guidelines [23] recommend the TUG as a clinically useful tool for assessing gait, strength, and balance in the risk of falls in elderly patients. A prospective cohort study in the UK revealed that TUG was significantly and independently correlated with the future falls of people aged 65 years and over [24]. The present study also revealed that TUG is suitable for the prediction of new falls in the elderly aged ≥80 years old in the community. A TUG result of < 10 s indicates a person is independently mobile, a TUG result of < 20 s means a person is mostly capable of independent activity, a TUG result of 20–29 s indicates instability, and a TUG result of > 30 s suggests some dyskinesia [16]. Studies concluded that a TUG result of ≥ 13.5 s could be used as an evaluation index of fall risk. However, a TUG result of 13.5 s has not been widely proven to be the most appropriate critical value. This study revealed that a TUG result of > 12 s increased the risk of falls by 98% in the elderly population enrolled in this study. Therefore, although TUG can be used to predict new falls in the elderly in the community, more research is needed to determine the critical value for future assessments.

The daily activity ability of elderly people is correlated with cognitive and psychological status. Fauth et al. showed that even after controlling for baseline cognitive status, ADL dysfunction was still an important predictor of dementia risk, and to a lesser extent, it was also affected by psychological and environmental factors. In this study, the median (quarterback interval) of ADL scores of the included samples is 100 (95, 100), which can initially exclude dementia [25], but not Mild Cognitive Impairment (MCI) [26]. The inclusion of tools such as the MINI-mental Status Exam, Home Fall HiHA (HIHA) and other kinds of psychological assessment will ensure the comprehensive assessment of fall risks for elderly people in the future.

Intrinsic capacity is defined as the resilience that an individual has to overcome a variety of environmental, physical and psychological factors. When a person declines at a more rapid rate than the age-related decline in intrinsic capacity, they are said to be exhibiting frailty [27]. Physical frailty was operationalized by Fried et al. [28], who defined it as being the presence of three or more of the following: fatigue, weight loss, weakness, slow walking gait and limited physical activity. Since frail persons are at a greater risk of falls, the assessment of frailty should also be added in future studies.

For the elderly, fragility fractures in themselves are not fatal but the underlying diseases and multiple system complications of the elderly are often the main causes of the high fatality rate. The 1-year mortality rate of elderly patients with hip fractures is 12–37%, and approximately half of the patients did not recover their independent living ability [18]. BMD alone has high specificity but low sensitivity in evaluating the risk of fractures. Therefore, BMD alone is not recommended for population screening [29]. Moreover, FRAX tools do not include fall factors in the calculation, and this may lead to an underestimation of the fracture risk of this population [30]. In the present study, the incidence of fragility fractures within 12 months was 10.0% and the main three fracture sites were the thoracolumbar spine, hip, and distal forearm. Female patients with an outdoor activity of < 30 min/day, a previous fragility fracture history, a high FRA score, new falls, low BMD, low muscle strength, low muscle function, and low balance function were more likely to have new fragility fractures. Previous studies have also revealed that female menopause was the most common cause of osteoporosis. Within 3–5 years of menopause, due to the lack of estrogen in the body, the bone mass of females will be rapidly lost, and this can cause osteoporosis or even fragility fractures [30]. Other diseases with a high incidence in the elderly, such as diabetes, rheumatic immune diseases, and liver and kidney dysfunction, will also affect bone metabolism, leading to a decrease in the total amount and quality of bones, and an increase in the likelihood of osteoporosis and fragility fractures [31]. The logistic regression analysis further revealed that new falls, low BMD of the lumbar vertebrae, and low walking speed were the significant risk factors of new fragility fractures. A previous study also revealed that the decreasing BMD of the lumbar vertebrae after 80 years of age was correlated to vascular calcification and joint degeneration in the lumbar vertebrae [32].

The strength of this study is that by analyzing the data, we have shown that evaluation tools that are inexpensive and simple to use, such as walking speed, TUG, ADL, and FRA, can be used to screen for falls and fragility fractures in the elderly population. However, limitations also exist, because as a common senile syndrome, falls have multiple potential risk factors. This study explored falls in the older population and preliminary screening tools for brittle fracture risk and focused on the functional assessment of physical activity, but it did not consider cognitive, psychological, social, or environmental factors, or medication. Further research will focus on screening out the physical activity of functional relation between loss and fall over and over again the crowd, multi-factor comprehensive assessment and mechanism research.

## Conclusion

This study has demonstrated that inexpensive and straightforward evaluation tools can be used to screen for high fall risk and fragility fractures in the elderly population. Subsequently, those at risk can be recommended to the hospital for further examination, individual intervention programs can be developed, and follow-up can be carried out in the community. The researchers hope to use these assessment methods to effectively reduce the incidence of falls and fragility fractures, and shorten the disability period of the elderly after experiencing a fall and fragility fracture, thereby improving their health and quality of life, and reducing the associated pressure on the family and the social care system.

## Data Availability

Not applicable.

## Abbreviations

BMD: Bone mineral density
COPD: Chronic obstructive pulmonary disease
ADL: Activity of Daily Living
FRA: Fall risk assessment
UGS: Usual gait speed
TUG: timed up & go
CRT: Chair rising test
BUN: Blood urea nitrogen
Cr: Creatinine
ALB: Albumin
PTH: Parathyroid hormone
T: Total testosterone
E2: Estradiol
P1NP: Type I amino-terminal propeptide
β-CTx: β-C-terminal telopeptide of type I collagen
OC: Osteocalcin
25OHD: 25-Hydroxyvitamin
DXAdual-energy: X-ray absorptiometry
WHO: World Health Organization
